# Imaging methods for quantifying glenoid and Hill-Sachs bone loss in traumatic instability of the shoulder: a scoping review

**DOI:** 10.1186/s12891-015-0607-1

**Published:** 2015-07-18

**Authors:** David J. Saliken, Troy D. Bornes, Martin J. Bouliane, David M. Sheps, Lauren A. Beaupre

**Affiliations:** Department of Surgery, University of Alberta, 6-110 CSB, 8440-112 Street NW, Edmonton, AB T6G 2B7 Canada; 2-50 Corbett Hall, Department of Physical Therapy, University of Alberta, Edmonton, AB T6G 2G4 Canada

**Keywords:** Shoulder instability, Diagnostic imaging, Specificity, Sensitivity

## Abstract

**Background:**

Glenohumeral instability is a common problem following traumatic anterior shoulder dislocation. Two major risk factors of recurrent instability are glenoid and Hill-Sachs bone loss. Higher failure rates of arthroscopic Bankart repairs are associated with larger degrees of bone loss; therefore it is important to accurately and reliably quantify glenohumeral bone loss pre-operatively. This may be done with radiography, CT, or MRI; however no gold standard modality or method has been determined. A scoping review of the literature was performed to identify imaging methods for quantifying glenohumeral bone loss.

**Methods:**

The scoping review was systematic in approach using a comprehensive search strategy and standardized study selection and evaluation. MEDLINE, EMBASE, Scopus, and Web of Science were searched. Initial selection included articles from January 2000 until July 2013, and was based on the review of titles and abstracts. Articles were carried forward if either reviewer thought that the study was appropriate. Final study selection was based on full text review based on pre-specified criteria. Consensus was reached for final article inclusion through discussion amongst the investigators. One reviewer extracted data while a second reviewer independently assessed data extraction for discrepancies.

**Results:**

Forty-one studies evaluating glenoid and/or Hill-Sachs bone loss were included: 32 studies evaluated glenoid bone loss while 11 studies evaluated humeral head bone loss. Radiography was useful as a screening tool but not to quantify glenoid bone loss. CT was most accurate but necessitates radiation exposure. The Pico Method and Glenoid Index method were the most accurate and reliable methods for quantifying glenoid bone loss, particularly when using three-dimensional CT (3DCT). Radiography and CT have been used to quantify Hill-Sachs bone loss, but have not been studied as extensively as glenoid bone loss.

**Conclusions:**

Radiography can be used for screening patients for significant glenoid bone loss. CT imaging, using the Glenoid Index or Pico Method, has good evidence for accurate quantification of glenoid bone loss. There is limited evidence to guide imaging of Hill-Sachs bone loss. As a consensus has not been reached, further study will help to clarify the best imaging modality and method for quantifying glenohumeral bone loss.

**Electronic supplementary material:**

The online version of this article (doi:10.1186/s12891-015-0607-1) contains supplementary material, which is available to authorized users.

## Background

Glenohumeral instability (GHI) has been associated with a recurrence rate ranging from 30-90 % [1–3]. Currently, arthroscopic Bankart repair using modern suture anchor techniques have failure rates ranging from 4-17 % [4–6]. A number of risk factors have been proposed to predict recurrence of GHI following arthroscopic Bankart repair including: age, humeral head and glenoid bone loss, shoulder hyperlaxity, and contact activity [1, 7–10]. Glenoid bone loss occurs in up to 90 % of patients with recurrent GHI [11] and, on average, occurs nearly parallel to the long axis of the glenoid (03:01–03:20 on a clock face) [12, 13]. Burkhart *et al.* showed that significant glenoid bone loss, approximately 25-45 % of glenoid width loss, was associated with higher failure rates of arthroscopic Bankart repairs [14, 15]. The critical defect size for predicting failure of arthroscopic Bankart repairs has been explored biomechanically [16–18]. Yamamoto *et al.* found that glenoid loss greater than 20 % glenoid length and 26 % of glenoid width, destabilized the shoulder [19]. The threshold of glenoid bone loss above which arthroscopic Bankart repairs may fail has generally been accepted as glenoid width loss ≥25 %, which is equivalent to ≥19 % of the glenoid length and ≥20 % of the surface area created by a best-fit circle on the inferior surface of the glenoid [14–20]. Width loss of 25 % may be expressed as a millimeter defect, varying based on individual glenoid anatomy but is approximately 6-8 mm given that the average glenoid width at the level of the bare area is 24-26 mm [19, 21]. It is important to keep in mind how one calculates glenoid bone loss, as the threshold for surface area is different than for glenoid width.

Humeral head bone loss, also known as a Hill-Sachs lesion, occurs in up to 93 % of patients with recurrent GHI [22]. Hill-Sachs lesions are oriented in the axial plane approximately at 07:58+/−00:48 or at an angle of 239.1+/−24.3 ° from 12 o’clock [23]. In a biomechanical study, Kaar *et al.* showed that defects created at an orientation of 209 ° significantly decreased resistance to dislocation when they were greater than 5/8 the depth of the radius of the humeral head in the axial plane [24]. Sekiya *et al.* demonstrated that Hill-Sachs lesions created in a posterolateral orientation benefited from allograft transplantation when greater than 37.5 % of the humeral head diameter [25]. In a later cadaveric study, Sekiya *et al.* showed that defects of 25 % of the humeral head diameter in isolation did not increase risk of dislocation following a capsulolabral repair [26]. There is a relationship between recurrent dislocations and failure of arthroscopic Bankart repair with increasing size of the Hill-Sachs lesion, although an accepted threshold value for Hill-Sachs bone loss has not yet been determined [9, 27, 28]. Hill-Sachs bone loss occurs simultaneously with glenoid bone loss in up to 62 % of GHI patients [29]. The way in which the glenoid and Hill-Sachs lesions interact and contribute to GHI is complex but does appear to be synergistic [30].

The degree of glenohumeral bone loss plays a role in surgical decision-making. The glenoid lesion may be treated successfully with an arthroscopic Bankart repair if it is smaller than the previously mentioned values. Larger glenoid bone lesions may require a bone augmentation procedure such as a Latarjet coracoid transfer or a J-graft procedure (bone graft harvested from iliac crest) [31]. A large Hill-Sachs defect can be addressed via a remplissage procedure (posterior capsulodesis and infraspinatus tenodesis), allograft, resurfacing arthroplasty, or rotational osteotomy. Ideally, the surgeon would be able to accurately quantify bone loss preoperatively to best ensure a successful post-operative outcome with the lowest rate of recurrent instability and the least amount of post-surgical morbidity.

**Fig. 1:**
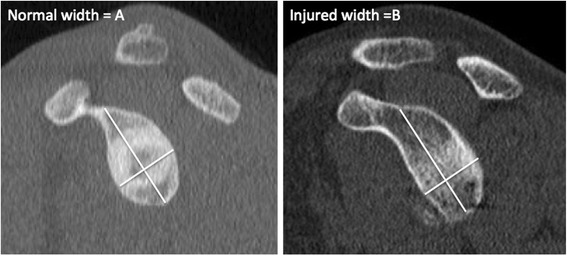
Griffith Index. Width measurements are made perpendicular to a line through the vertical axis of the glenoid and compared to the uninjured glenoid (B/A x 100) to determine percent width loss (adapted from Griffith *et al.* [33])

**Fig. 2:**
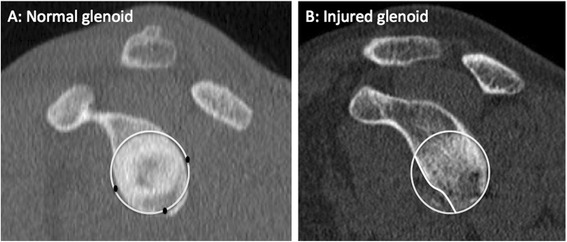
Pico Method. The original description of Pico Method involved determining the circumference of the contralateral, normal inferior glenoid circle based on the intact 3–9 o’clock margin, transferring the circle to the injured glenoid, and manually tracing out the glenoid defect and using software to calculate surface area bone loss. Note that the Pico Method has also been used with the intact 6 o’clock-9 o’clock postero-inferior margin of the injured glenoid to determine the pre-injury glenoid circle (adapted from Bois *et al.* [63])

Multiple imaging methods exist for quantifying glenoid and Hill-Sachs bone loss including radiography, computerized tomography (CT), and magnetic resonance imaging (MRI). Radiography is inexpensive and easy to obtain but may be less accurate in detecting the presence of bone lesions compared to CT. Radiographic methods have been proposed using both basic views (true AP, axillary) and special views (Bernageau profile) to measure glenoid and Hill-Sachs bone loss. CT is easy to obtain and accurate with respect to bony detail, but necessitates radiation exposure. MRI is expensive and more difficult to obtain in public health care systems but has no radiation exposure, and may provide information on associated soft tissue lesions involving the labrum, rotator cuff, and capsule. However, assessment of bone lesions may be inferior to that of CT. Two common methods of calculating glenoid bone loss using CT and MRI are width measurements such as the Griffiths Index (Fig. 1) and best-fit circle surface area measurements such as the Pico Method (Fig. 2) [32, 33]. These methods involve reformatting to subtract the humeral head and get an *en face* view of the glenoid. The inferior 2/3 of the glenoid approximates a true circle, the size of which can be estimated based on either the contralateral glenoid or intact posteroinferior margins of the injured glenoid [11, 34, 35]. Bone loss can then be expressed as the area lost from the circle or the anterior-to-posterior width loss. Determination for Hill-Sachs bone loss has included, among others, depth, width, and length measurements [36–39].

The degree of glenohumeral bone loss affects the success of arthroscopic Bankart repair and, at present, there is no consensus on a gold standard imaging method or modality for the quantification of glenohumeral bone loss. We performed a scoping review of the literature to identify current published imaging methods for quantifying glenoid and humeral head bone loss in GHI and to evaluate if there was a gold standard method and modality supported by evidence.

## Methods

### Study design

A scoping review was performed to evaluate the literature based on established guidelines [40, 41]. Scoping reviews are designed to assess the extent of a body of literature and identify knowledge gaps. Although qualitative in nature, the review can be systematic in approach through a comprehensive search strategy and standardized study selection and evaluation, as in our study. Due to heterogeneity in the articles reviewed, no meta-analyses were performed in this study.

### Selection criteria

Studies were included if the following conditions were met: (1) publication after the year 2000 (following a preliminary review of the literature, the majority of relevant imaging methods were published after this time point; publications prior to 2000 are included in our introduction and discussion when historically relevant); (2) use of human or cadaveric human subjects; (3) evaluation of imaging methods including radiography, CT, and/or MRI; and (4) quantification of glenoid or Hill-Sachs bone loss using these imaging modalities. Criteria for exclusion were: (1) non-English language; and (2) publication in the form of an abstract, letter, or review article.

### Search strategies

MEDLINE, EMBASE, Scopus, and Web of Science were searched from January 2000 until July 2013. A search algorithm was created with the guidance of a medical librarian (see Additional file 1).

### Study selection

Article selection was performed over two rounds, by two orthopaedic surgery residents with the assistance of two upper extremity fellowship-trained orthopaedic surgeons. During the first round, selection was based on the review of titles and abstracts. To be as inclusive as possible, an article was carried forward to the next stage if either reviewer thought that the study was appropriate. Final study selection was based on full text review using the aforementioned inclusion and exclusion criteria. Duplicate studies were kept until the final article selection. Consensus was reached for final article inclusion through discussion amongst the investigators.

### Data extraction

One reviewer extracted study design, imaging modality evaluated, patient characteristics, quantification method used, and findings. A second reviewer independently assessed data extraction for any discrepancies. When provided by the authors, the reliability, accuracy, sensitivity, and specificity are presented in the results section with accompanying tables.

## Results

### Article selection

**Fig. 3:**
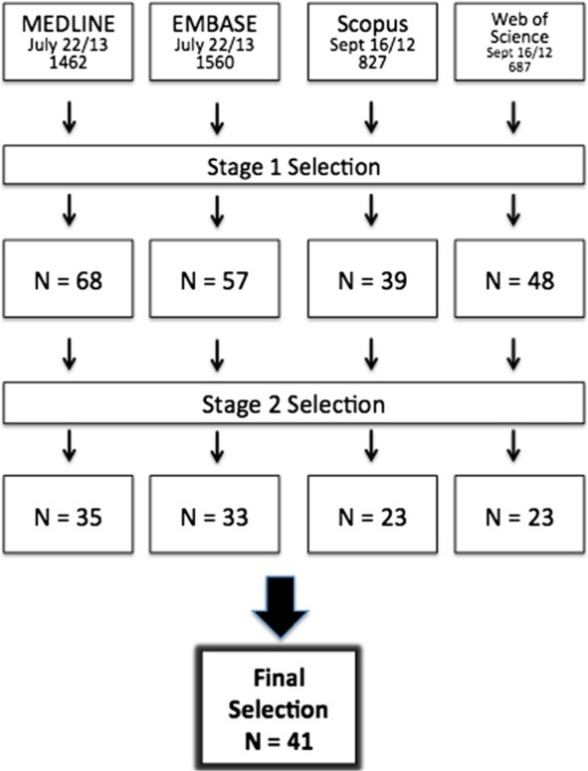
Flowchart of Study Selection

Initial literature search retrieved 4536 total articles: 1462 from MEDLINE, 1560 from EMBASE, 827 from Scopus, and 687 from Web of Science (Fig. 3). After the initial review of titles and abstracts, 212 articles were retained. Following review of the full text, 114 articles remained. After the removal of duplicates, 41 articles were included.

### Article summary

Tables 1, 2, 3, 4, 5 and 6 summarize the selected articles. We retained 11 articles focusing on Hill-Sachs bone loss, 32 for glenoid bone loss, and 2 articles evaluated both. There were a significantly higher number of articles evaluating CT imaging (38) compared to radiography (11) and MRI (10). For glenoid bone loss, radiography was evaluated in 7 studies [18, 42–47], MRI in 8 studies [21, 42, 44, 48–53], and CT in 32 studies [11, 18, 20, 21, 32, 33, 35, 42–51, 54–69]. For Hill-Sachs bone loss, radiography was evaluated in 5 studies [37–39, 46, 47], MRI in 2 studies [70, 71], and CT in 5 studies [23, 36, 72–74].

**Table 1:** Studies Assessing Glenoid Bone Loss with Radiography

Study	Modality	Details	Quantification Technique	Findings
Charousset *et al.* [47]: Retrospective case series	Radiography; 2DCT	31 patients	***True AP radiography:***	***Loss of sclerotic line (ICC):***
		***Assessment:***	Loss of sclerotic line	Inter-observer 0.44-0.47
		True AP view; 2DCT arthrogram; 3 observers measured twice	***CT***:	Intra-observer 0.66-0.93
		***Outcome:***	Griffith Index (Fig. 1); best-fit circle width loss (Fig. 10)	***Griffiths Index (ICC):***
		Reliability		Inter-observer 0.68-0.71
				Intra-observer 0.78-0.90
				***Best-fit circle width loss (ICC)***:
				Inter-observer 0.74
				Intra-observer 0.90-0.95
Itoi *et al.* [18]: Cadaveric study	Radiography; 2DCT	12 cadavers	***Radiography***:	***21 % glenoid length defect:***
		***Assessment:***	West Point & axillary views	18.6 % on West Point view 2.3 % on axillary view
		45 ° angle defects created at 0, 9, 21, 34, & 46 % of glenoid length; radiography at each cut; 1 observer measured twice	***CT:***	50 % loss of width on CT
		***Outcome***:	Width of the inferior ¼ of the glenoid measured in a single axial slice	
		Correlation, reliability		***Correlation coefficients***:
				0.905-0.993
				***Coefficients of variance***:
				0.5-3.6 %
Jankauskas *et al.* [45]: Retrospective case–control study	Radiography; 2DCT	86 patients	Superoinferior length of bone defect	***Detecting bone lesion:***
		***Assessment:***		Sensitivity 54-65 %
		True AP radiography; 2 observers on radiography; 1 observer on CT		Specificity 100 %
		***Outcome:***		Inter-rater reliability: kappa = 0.88
		Reliability; sensitivity; specificity		***Radiography*** ***vs.*** ***CT:***
				9 shoulders with mean 8.2 ± 3.5 mm glenoid bone loss on CT were missed on radiography
Sommaire *et al.* [46]: Retrospective cohort study	Radiography; 2DCT	77 patients	***Radiography:***	***Radiographic D1/D2 ratio (p = 0.003):***
		***Assessment:***	Bernageau view of both shoulders to calculate D1/D2 ratio (Fig. 4)	4.2 % patients without recurrence
		Pre-operative Bernageau radiographs & 2DCT of unilateral shoulder before arthroscopic Bankart repair; 1 observer measured once	***CT:***	
		***Outcome:***	Gerber‘s X index (Fig. 7)	5.1 % in patients with recurrence
				***CT:***
				Recurrence Rate (p = 0.004):
				Gerber X index < 40 % =20 %
		Need for revision correlated with imaging		Gerber X Index >40 % =12.7 %
				***Note:*** Reliability not assessed
Murachovsky *et al.* [43]: Prospective case–control study	Radiography; 3DCT	10 patients; 50 healthy subjects	***Radiography:***	***Reliability:***
		***Assessment:***	Bernageau view (D1/D2) ratio (Fig. 4)	Intra-observer ICC 0.897-0.965
		Bilateral radiography (all subjects) & CT (instability subjects); 1 radiologist measured CT; 3 orthopaedic surgeons measured 3 times each	***3DCT:***	Inter-observer ICC 0.76-0.81
		***Outcome:***	Glenoid AP width measured bilaterally to calculate % bone loss	Difference between radiography & CT non-significant (2.28 %)
		Reliability		

*List of Abbreviations:* *ICC* intraclass correlation coefficient; *PE* percent error

**Table 2:** Studies Assessing Glenoid Surface Area Loss with CT and MRI

Study	Modality	Details	Quantification Technique	Findings
Barchilon *et al.* [20]: Prospective case series	2DCT; 3DCT	13 patients	***Best-fit circle surface area:***	***Method 1 with method 2:***
		***Assessment:***	Approximation based on intact posteroinferior edge of ipsilateral glenoid	R^2^ = 0.91
		2DCT & 3DCT using 3 methods	***(1)*** Software directly measured area of circle and area of missing area using 2DCT (gold standard)	***Method 1 with method 3:***
		***Outcome:***	***(2)*** Mathematical formula to calculate % surface area loss using 2DCT based on circle radius & defect depth with software	R^2^ = 0.60
		Intra-method comparison	***(3)*** Manually measured defect depth & circle radius using 3DCT & femoral head gauge; formula to calculate % surface area	***Note*** **:** BCSA methods can be applied without computer software
Hantes *et al.* [65]: Cadaveric study	3DCT	14 cadavers	***Best-fit circle surface area:***	***Reliability:***
		***Assessment:***	Sugaya Method	Coefficient of variation 2.2-2.5 %
		CT scan following 3 serial osteotomy’s; 1 observer measured 5 times for 2 glenoids		
		***Outcome:***		
		Reliability		
Huijsmans *et al.* [21]: Cadaveric study	3DCT; MRI	14 cadavers	***Best-fit circle surface area:***	***Difference with digital picture:***
		***Assessment:***	Circle approximated based on ipsilateral glenoid; software used	CT −0.81 % to −1.21 %
		Digital picture, CT, & MRI before/after osteotomy (random size) on anterior glenoid; 2 observers measured 3 times		MRI 0.61 % to 0.74 % (non-significant)
		***Outcome:***		***CT:***
		Reliability		Inter-observer r^2^ = 0.94
				Intra-observer r^2^ = 0.97 (observer 1) and 0.90 (observer 2)
				***MRI:***
				Inter-observer r^2^ = 0.87
				Intra-observer r^2^ = 0.93 (observer 1) and r^2^ = 0.92 (observer 2)
				***Digital image:***
				Inter-observer r^2^ = 0.97
Lee *et al.* [52]: Prospective case series	2DCT; MRI	65 patients	***1)*** ***Best-fit circle surface area (Pico method)***	***Inter-observer ICC:***
		***Assessment:***	***2) Best-fit circle width method***	0.95 for best-fit circle width
		CT (bilateral) & MRI followed by arthroscopy; 1 observer measured CT once; 3 observers measured MRI once; 1 observer measured MRI 3 times	Arthroscopy with bare-area technique (used as gold standard)	0.90 for area (Pico method)
		***Outcome:***		***Intra-observer reliability ICC:***
		Reliability		0.98 width
				0.97 area
				***Correlation:***
				CT & MRI 0.83
				CT & arthroscopy 0.91
				MRI & arthroscopy 0.84
Magarelli *et al.* [32]: Prospective case series	2DCT	40 patients	***Best-fit circle surface area method:***	***Intra-observer reliability:***
		***Assessment:***	Pico method based on contralateral glenoid	ICC 0.94
		Bilateral CT; 1 observer measured 3 times; 1observer measured once		SEM 1.1 %.
		***Outcome:***		***Inter-observer reliability:***
		Reliability		ICC 0.90
				SEM 1.0 %.
				***Note:*** No comparison to other methods
Magarelli *et al.* [57]: Prospective cohort study	2DCT; 3DCT	100 patients	***Best-fit circle surface area:***	***Mean difference:***
		***Assessment:***	Pico method based on contralateral glenoid	0.62 %+/−1.96 %
		Bilateral CT; 2 observers measured once		***Note:*** No reliability measurement
		***Outcome:***		
		Agreement between 2D & 3D CT		
Nofsinger *et al.* [35]: Retrospective case series	3DCT	23 patients	***Best-fit circle surface area:***	***Normal shoulder:***
		***Assessment:***	Anatomic Glenoid Index: circle matched to postero-inferior glenoid of contralateral glenoid; software measured area of circle	Circle fit true glenoid closely −100.5 %, SD 2.2 %.
		Bilateral pre-op CT followed by surgical repair (12 Bankart, 11		***Mean AGI for Bankart group:***
		Latarjet); 3 blinded observers measured once	(A1); circle manually adjusted to fit defect & area again calculated by software (A2); area loss = A2/A1 x 100	92.1 %+/−5.2 %
		***Outcome:***		***Mean AGI for Latarjet:***
		Surgical decision based on size >25 % at arthroscopy; reliability		89.6 %+/−4.7 %
				***Inter-rater reliability (Pearson correlation coefficient):***
				0.60-0.84
				***Note:*** Did not have the power to separate the two surgical groups
Park *et al.* [60]: Retrospective case series	2DCTA	30 patients	***Best-fit circle surface area:***	***Intra-observer reliability:***
		***Assessment:***	Pico method based on ipsilateral glenoid	ICC 0.96-1.00;
		CTA taken pre-op, at 3 months, and 1 year after bony Bankart repair; 1 observer measured 6 times		Positive relationship between number of dislocations & defect size
		***Outcome:***		
		Reliability		
Sugaya *et al.* [11]: Case–control study	3DCT	100 patients, 10 healthy volunteers	***Best-fit circle surface:***	Normal glenoid did not differ significantly from contralateral glenoid; inferior portion of glenoid approximates a true circle; did not compare measurements to arthroscopic measurements; no reliability measurements
		***Assessment:***	Sugaya Method with bone fragment manually outlined	***Note*** **:** Technique would not work in case of attritional bone loss without a Bankart fragment
		Bilateral CT; defects categorized as: small (<5 %), medium (5-20 %), or large (>20 %); patients also had arthroscopy: 1 observer measured once		
		***Outcome:***		
		Comparison to normal glenoid		

*List of Abbreviations:* ICC: intraclass correlation coefficient; PE: percent error; SEM: standard error of measurement; R^2^: coefficient of determination; AGI: anatomic glenoid index

**Table 3:** Studies Assessing Glenoid Width Loss with CT and MRI

Study	Modality	Details	Quantification Technique	Findings
Charousset *et al.* [47]: Retrospective case series	Radiography; 2DCT	31 patients	***True AP radiography:***	***Loss of sclerotic line:***
		***Assessment:***	loss of sclerotic line	Inter-observer ICC 0.44-0.47
		True AP radiography & 2DCT arthrogram; 3 observers measured twice	***CT:***	Intra-observer ICC 0.66-0.93
		***Outcome:***	Griffiths Index (Fig. 1) & best-fit circle width loss (Fig. 10)	***Griffiths Index:***
		Reliability		Inter-observer ICC 0.68-0.71
				Intra-observer ICC 0.78-0.9
				***Best-Fit Circle Width Loss***:
				Inter-observer ICC 0.74
				Intra-observer ICC 0.9-0.95;
Chuang *et al.* [68]: Retrospective case series	3DCT	25 patients	***CT:***	Glenoid Index correctly categorized 96 % of patients
		***Assessment:***	Glenoid Index (Fig. 5)	***Glenoid Index***:
		Bilateral 3DCT followed by diagnostic arthroscopy: >25 % glenoid width loss (Latarjet); <25 % glenoid width loss (arthroscopic Bankart)	***Arthroscopy:***	Latarjet group: mean 0.668
		***Outcome:***	Bare area method	Bankart group: mean 0.914
		Ability to predict type of surgery offered		
Griffith *et al.* [33]: Case–control study	2DCT; 3DCT	40 patients (46 shoulders); 10 healthy subjects	***Measurements:***	***Healthy subjects***:
		***Assessment:***	Width & cross-sectional surface area on axial slice; length; width; length:width ratio; glenoid surface area by point tracing; flattening of anterior glenoid curvature	No significant difference in side-side measurements
		Bilateral CT;1 observer measured once		***Instability Subjects:***
		***Outcome:***		Width (3 mm difference; 10.8 % width loss); length:width ratio, & cross-sectional area significantly different side-to-side
		Glenoid comparison with healthy subjects on *en face* glenoid view		
Griffith *et al.* [58]: Prospective case series	2DCT	50 patients	***Width Measurement:***	***CT correlation with arthroscopy:***
		***Assessment:***	Griffiths Index (Fig. 1)	Pearson Correlation Coefficient r = 0.79
		Bilateral CT followed by arthroscopy; compared to measurements made during arthroscopy (bare spot method); 1 observer measured once		
		***Outcome:***		Sensitivity 92.7 %
		Correlation, PPV, NPV		Specificity 77.8 %
				PPV 95 %; NPV 70 %.
				***Mean bone loss***(p = 0.17):
				CT 11.0 %+/−8.1 %
				Arthroscopy 12.3 %+/−8.8 %
Griffith *et al.* [62]: Case–control study	2DCT	218 patients; 56 healthy subjects	***Width measurement:*** Griffith Index (Fig. 1)	Normal side-to-side glenoid width difference small (0.46 mm);
		***Assessment:***	***Note*** **:** Glenoid bone loss not calculated on bilateral subjects	***Reliability:***
		Bilateral CT; 1 observer measured all subjects; 2 observers measured 40 patients twice		Inter-observer reliability ICC 0.91
		***Outcome:***		Intra-observer reliability ICC 0.95
		Reliability		
Gyftopoulos *et al.* [48]: Cadaveric study	2DCT; 3DCT; MRI	18 cadavers	***Width method:***	***Intra-observer concordance correlation coefficient (CCC):***
		***Assessment:***	Best-fit circle width method based on ipsilateral glenoid	2DCT 0.95
		Defects created along anterior and antero-inferior glenoid; 3 observers measured defect size once; 1 observer re-measured at 4 weeks; gold standard was digital photograph		3DCT 0.95
		***Outcome:***		MRI 0.95
		Reliability, PE		***Inter-observer CCC:***
				2DCT −0.28-0.88
				3DCT 0.82-0.93
				MRI 0.70-0.96
				***Percent error:***
				2DCT 2.22-17.11 %
				3DCT 2.17-3.50 %
				MRI 2.06-5.94 %
Lee *et al.* [52]: Prospective cohort study	2DCT; MRI	65 patients	***1) Best-fit circle surface area:***	***Inter-observer reliability (ICC)***
		***Assessment:***	Pico Method	Best-fit circle width R = 0.95
		CT (bilateral) & MRI followed by arthroscopy; 1 observer measured CT once; 3 observers measured MRI once; 1 observer measured MRI 3 times;arthroscopy was gold standard using bare-area technique	***2) Best-fit circle width method***:	Area (Pico method) R = 0.90
		***Outcome:***	Based on contralateral glenoid	***Intra-observer reliability:***
		Reliability, correlation		Width R = 0.98, area R = 0.97
				***Correlation:***
				CT-MRI r = 0.83
				CT-arthroscopy r = 0.91
				MRI-arthroscopy r = 0.84
Moroder *et al.* [50]: Retrospective case series	3DCT, MRI	48 patients	***Width method:***	***CT for glenoid lesion:***
		***Assessment:***	Best-fit circle width method	Sensitivity 100 %
		Pre-op CT & MRI evaluated after failed instability surgery; findings at initial operation were comparators; 1 observer measured significant glenoid defects (>20 % of width)		Specificity 100 %
		***Outcome:***		***MRI for significant lesion:*** Sensitivity 35.3 %
		Sensitivity, specificity		Specificity 100 %
				CT would have misled treatment in only 4.2 %
Tian *et al.* [51]: Prospective cohort study	2DCT; MRA	41 patients; 15 control patients	***Width method:***	No significant size measurements between MRA (10.48 %+/−8.71 %) & CT (10.96 %+/−9.0 %; p = 0.288).
		***Assessment:***	Best-fit circle width method based on ipsilateral glenoid (Fig. 10)	***Correlation between methods:***
		CT & MRA; 2 observers measured once		Pearson correlation coefficient r = 0.921; SD 3.3 %
		***Outcomes:***		
		Correlation		

*List of Abbreviations:* ICC: intraclass correlation coefficient; PE: percent error; PPV: positive predictive value; NPV: negative predictive value

**Table 4:** Studies Directly Comparing Imaging Methods for Assessing Glenoid Bone Loss

Study	Modality	Details	Quantification Technique	Findings
Bishop *et al.* [42]: Cadaveric study	Radiography; 2DCT; 3DCT; MRI	7 cadavers	Observers measured bone loss using his/her usual approach (Methods not specified)	***Overall agreement with gold standard (kappa score):***
		***Assessment:***		3DCT 0.5
		Serial imaging of shoulder after osteotomies of 0 %, <12 %, 12-25 %, 25-40 %; manually measured glenoid width through bare area using a digital caliper (gold standard); 12 observers measured twice		CT 0.4
		***Outcome:***		MRI 0.28
		Reliability		Radiography 0.15
				***Intra-observer reliability (kappa):***
				3DCT 0.59
				CT 0.64
				MRI 0.51
				Radiography 0.45
				***Note:*** 3DCT highest agreement & 2nd highest intra-observer reliability; radiography lowest agreement & reliability
Bois *et al.* [63]: Laboratory study	2DCT; 3DCT	Sawbones:1 model for anterior defect; 1 model for anteroinferior defect	***2DCT & 3DCT:***	***2D CT methods (ICC, PE):***
		***Assessment:***	Indicators: linear width/length (W/L) ratio; defect length; quantifiers: glenoid index (injured glenoid inferior circle diameter relative to uninjured glenoid diameter)	Defect length: 0.81, 7.68
		Osteotomies made at 0, 15 %, and 30 % of inferior glenoid circle diameter; gold standard measurement (3D laser scanner of model); 6 observers measured all 7 techniques	***3DCT:***	W/L ratio: 0.50, −16.34
		***Outcome:***	Quantifiers: linear ratio (d/R; d = radius to defect, R = circle radius); Pico method (3 variations):	Glenoid index, 0.3, −4.13
		Reliability, PE	***(1)*** Original circle method	***3D CT (ICC, PE):***
			***(2)*** Based on contralateral normal glenoid circle with 3 points of reference	Defect length: 0.90, 0.29
			***(3)*** Based on remaining intact glenoid cortex	W/L ratio: 0.88, −2.41
				Glenoid index: 0.69, 0.01 (0.85, 3.39 with other software platform)
				Linear ratio: 0.97, 29.9
				Pico (1): 0.98, 4.93
				Pico (2): 0.84, 7.32
				Pico (3): 0.86, 12.14
				***Note*** **:** Pico method (1) based on the contralateral, intact glenoid and Glenoid Index on 3DCT were most reliable & accurate; Glenoid Index on 2DCT was deemed invalid
Rerko *et al.* [44]: Cadaveric study	Radiography; 2DCT; 3DCT; MRI	7 cadavers	Observers measured bone loss using his/her usual approach (Methods not specified)	***Accuracy (PE):***
		***Assessment:***		3DCT −3.3 %+/−6.6 %
		Serial imaging of shoulder with osteotomies grouped as 0 %,<12 %, 12, 25 %, 25-40 %; gold standard defined as glenoid width using digital caliper; 2 radiologists & 2 orthopaedic surgeons measured twice		2DCT −3.7 %+/−8.0 %
		***Outcome:***		MRI −2.75 %+/−10.6 %
		PE, reliability		Radiography −6.9 % +/− 13.1 %
				***Intra-observer reliability (ICC):***
				3DCT 0.947
				2DCT 0.927
				MRI 0.837
				Radiography 0.726
				***Inter-observer reliability (ICC):***
				3DCT 0.87-0.93
				2DCT 0.82-0.89
				MRI 0.38-0.85
				Radiography 0.12-0.53

*List of Abbreviations:* ICC: intraclass correlation coefficient; PE: percent error

**Table 5:** Other Methods for Assessing Glenoid Bone Loss

Study	Modality	Details	Quantification Technique	Findings
De Filippo *et al.* [66]: Cadaveric study	2DCT	10 cadavers	***De Filipo Method***: Fig. 6	***Curved MPR CT:***
		***Assessment:***	***Note*** **:** Curved MPR assessed curved structures very accurate	PE 1.03 %; inter-observer reliability (Cronbach alpha) 0.995
		2 had anteroinferior defects created; 1 re-measured at 3 months; measured glenoid bone area on flat MPR & curved MPR of all 10 cadavers; laser scanner used directly on cadavers as gold standard; 3 radiologists measure once		Intra-observer reliability (ICC) 0.998
		***Outcome:***		***Flat MPR CT:***
		PE, reliability		PE 16.99 %
				Inter-observer reliability (Cronbach alpha) 0.995
				***Note:*** Authors conclude curved gives more accurate glenoid contour
Diederichs *et al.* [59]: Cadaveric study	3DCT	5 cadavers; 30 patients with no glenoid injury	Manually traced out border of glenoid; volume and surface area calculated with measurements made manually (to calculate volume, depth was assumed to be 10 mm)	***Coefficient of variation:***
		***Assessment:***		Width 1.7 %
		Glenoid width, height, surface area, & volume; osteotomy created on one cadaveric glenoid; compared to contralateral for calculation; 1 investigator measured study group; another measured the controls		Volume 1.3 %
		***Outcome:***		
		Coefficient of varaition		
Dumont *et al.* [49]: Technique description	CT; MRI	Authors describe a new method to calculate surface area loss	Best-fit circle to inferior glenoid; measured angle (alpha) from center of circle between superior and inferior edges of lesion; converted measured angle to percentage area loss = [(alpha-sinalpha)/2π] x 100	No assessment of reliability or comparison to other methods
				***Note:*** This method avoids issues with defect orientation and is simple to apply without complicated software
Tauber *et al.* [56]; Retrospective case series	CT	10 patients with associated glenoid fracture (>21 % glenoid length)	Fit circle to outer glenoid, measured glenoid length at 45° angle (A), measured length to defect (B); calculated bone loss as: (A x 0.965 – B)/A x 100	***Inter-observer reliability:***
		***Assessment:***		ICC = 0.81
		2 examiners measured once		Average width loss 26.2 %
		***Outcome:***		
		Reliability		
Van Den Bogaert *et al.* [69]: Cadaveric study	2DCT	20 cadavers	***Glenohumeral index:***	***Glenohumeral Index Compared to Gold Standard:***
		***Assessment:***	Maximal AP diameter of humeral head / maximal AP diameter of glenoid (axial images)	Non-significant difference
		Diameter measured with a digital caliper in vitro (gold standard) followed by CT quantification; 3 observers measured once		
		***Outcome:***		
		Direct comparison		

*List of Abbreviations:* ICC: intraclass correlation coefficient

**Table 6:** Studies Assessing Hill-Sachs Bone Loss with Radiography, CT and MRI

Citation	Method	Details	Quantification Method	Findings
Charousset *et al.* [47]: Retrospective case series	Radiography	26 patients	***Quantitative assessment:***	***P/R ratio reliability:***
		***Assessment:***	P/R ratio on true AP radiography in internal rotation (Fig. 10)	Inter-observer ICC 0.81-0.92
		3 observers measured twice	***Qualitative assessment:***	Intra-observer ICC 0.72-0.97
		***Outcome:***	True AP radiograph in external rotation (present or absent lesion)	***Qualitative assessment reliability:***
		Reliability		Inter-observer ICC 0–0.30
				Intra-observer ICC 0.06-0.92
				***Note:*** Simple patient positioning and reliable
Ito *et al.* [38]: Retrospective case series	Radiography	27 patients (30 shoulders)	***Width and depth of Hill-Sachs lesion measured:***	***Width difference*** ***(p > 0.05)*** ***:***
		***Assessment:***	Supine position; arm 135 ° flexion, 15 ° internal rotation; radiography beam perpendicular	Dislocation group 13.4 mm+/−2.5 mm
		Divided into 2 groups: dislocation (11) and dislocation with recurrent subluxation (19); 1 observer measured once	***Note:*** Patient positioning may be cumbersome and difficult to replicate in a clinical setting	With subluxation group 13.8+/−3.5 mm
		***Outcome:***		***Depth difference (p < 0.05):***
		Width difference		Dislocation group 3.9+/−0.9 mm
				With subluxation group 2.1+/−1.0 mm
				***Note:*** Deeper lesions associated with subjective joint laxity but not number of dislocations
Kralinger *et al.* [39]: Retrospective cohort study	Radiography	166 patients	***Hill-Sachs Quotient:***	***Recurrence rate associated with Hill-Sachs Quotient:***
		***Assessment:***	Bernageau view and AP view at 60 ° internal rotation (Fig. 8)	Grade I 23.3 %
		1 observer measured once		Grade II 16.2 %
		***Outcome:***		Grade III 66.7 %
		Recurrence rate		
Sommaire *et al.* [46]: Retrospective cohort study	Radiography	77 patients	***d/R ratio***:	***Risk of recurrence (p = 0.016):***
		***Assessment:***	True AP radiograph in internal rotation (similar to Charousset *et al.* [2010]; Fig. 9)	9.6 % in d/R ratio <20 %
		Final clinical outcome after arthroscopic Bankart repair and imaging; 1 observer measured once		40 % in d/R ratio >20 %
		***Outcome:***		***Note:*** d/R ratio predictive of failure of arthroscopic Bankart repair
		Need for revision repair		
Hardy *et al.* [37]: Retrospective cohort study	Radiography; 2DCT	59 patients	***Radiograph 45 ° internal rotation view:***	***d/R ratio (p < 0.01)***:
		***Assessment:***	Depth of defect/radius of humerus (d/R) ratio (similar to Charousset *et al.* [2010])	Good/excellent group: 16.2 %
		After arthroscopic stabilization divided into 2 groups based on Duplay clinical functional score: good/excellent (38) fair/poor (21); 1 observer measured all patients once; 10 observers measured 10 patients	***CT:***	Poor/fair group: 21.3 %
		***Outcome:***	Humeral head radius (best-fit circle to circumference); defect width; defect depth (from edge of circle); defect length (amount of CT slices with the defect); lateralization angle (compared to AP line through center of head)	***Mean volume of lesion (p < 0.001):***
		Correlation of clinical score with radiographic findings; surgical failure rate	***Note:*** Radiographic technique easily obtained	Good/excellent group: 640 mm^3^
				Poor/fair group: 2160 mm^3^
				***Surgical failure rate:***
				d/R >15 %: 56 %
				d/R < 15 %: 16 %
				Presence of lesion, depth, lateralization angle, lesion, and humeral head volume ratio all non-significant between groups
				***Reliability*** ***:***
				Inter-observer reliability for depth and radius measurements non-significant
Kodali *et al.* [72]: Laboratory study	2DCT	6 anatomic bone substitute models	***Circle fit to humeral head:***	***Inter-observer reliability ICC:***
		***Assessment:***	Width and depth measured on sagittal, axial, and coronal planes (similar to Saito *et al.* (2009)	Depth - 0.879
		Circular humeral head defects created; 2DCT width-depth measurements made in 3 planes and compared to the defect sizes measured by a 3D laser scanner		Width 0.721
		***Outcome:***		***Accuracy (PE):***
		5 observers measured once		Width: sagittal 10.9+/−8.6 %, axial 10.5+/−4.4 %, coronal 15.9+/−8.6 %;
				Depth: sagittal 12.7+/−10.0 %, axial 16.7+/−10.2 %,coronal 22.5+/−16.6 %
Saito *et al.* [12]: Retrospective case-controls study	2DCT	35 patients; 13 normal	***Circle fit to the humeral head on axial slices***:	***Mean size of Hill-Sachs lesion:***
		***Assessment:***	Depth: greatest length of distance from floor of defect to edge of circle; width: measured between edges of defect	Depth 5.0+/−4.0 mm; width 22+/−6 mm
		1 observer measured 3 times		***Intra-observer reliability:***
		***Outcome:***		Pearson correlation coefficient: 0.954-0.998
		Reliability		Coefficient of variation: 0–7.4 %.
Cho *et al.* [36]: Prospective cohort study	3DCT	104 patients (107 shoulders)	***Fit circle to articular surface of humeral head:***	***Inter-observer reliability:***
		***Assessment:***	Axial and coronal planes: width and depth measured on axial and coronal slice where lesion was largest	ICC 0.629-0.992
		evaluated size, orientation, & location as means to predict engagement; engagement defined arthroscopically; 1 observer measured 27 randomly selected shoulders 3 times; 2nd observer measured once		***Intra-observer reliability:***
		***Outcome:***		ICC 0.845-0.998
		Reliability, size of Hill-Sachs lesion relationship to engaging lesions		***Size of Hill-Sachs lesion (axial):***
				Engaging group width 52 % & depth 14 %
				Non-engaging group width 40 % & depth 10 % (both p <0.001)
				***Size of Hill-Sachs lesion (coronal):***
				Engaging group width 42 % & depth 13 %
				Non-engaging group width 31 %, & depth 11 % (p = 0.012 & 0.007 respectively).
				***Note:*** Orientation of Hill-Sachs angle significantly higher in engaging lesions
Kawasaki *et al.* [73]: Modeling	3DCT	Evaluated 7 CT scans of bilateral shoulders	Created 3D contour; mirrored the normal shoulder and overlap contours; computer measured defect difference	Proposed a method to calculate humeral head bone loss
Kirkley *et al.* [70]: Prospective case series	MRI	16 patients	Hill-Sachs lesions were categorized as small (<1 cm) or large (>1 cm);	***Presence*** ***vs.*** ***absence of Hill-Sachs lesion:***
		***Assessment:***	***Note*** **:** Did not clarify slice or dimensions measured to determine Hill-Sachs lesion size	Kappa = 1
		MRI followed by arthroscopic evaluation; 2 observers measured once		***Distinguishing small from large lesion:*** Kappa = 0.44
		***Outcome:***		Not able to accurately quantify size
		Reliability		
Salomonsson *et al.* [71]: Prospective cohort study	MRI	51 patients	***Hill-Sachs depth***:	***Size of Hill-Sachs lesion:***
		***Assessment:***	Measured on axial slice at largest point	Stable group 5 mm; unstable group 3 mm (non-significant)
		MRI immediately and clinical follow-up to 105 months; divided into stable and unstable (recurrent instability); 2 observers measured once		
		***Outcome:***		
		Size of Hill-Sachs lesion correlation with recurrent instability		

*List of Abbreviations:* ICC: intraclass correlation coefficient; PE: percent error

### Glenoid imaging

#### Radiography

**Fig. 4:**
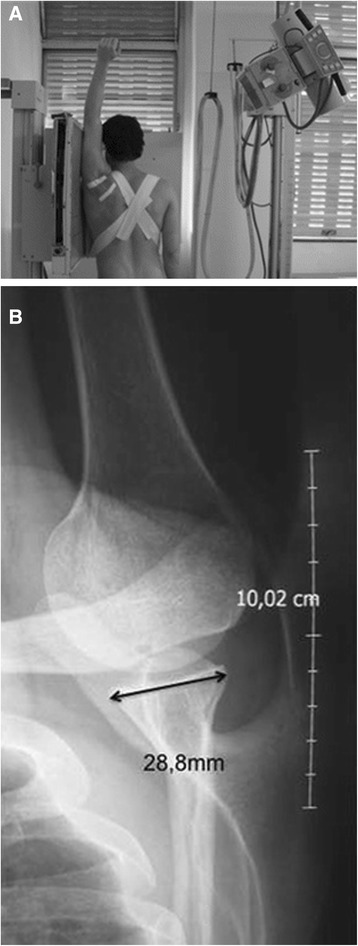
Bernageau Radiography. **a)** Patient positioning**. b)** Antero-posterior glenoid width measurement on this view (Murachovsky *et al.* [43])

Routine radiographic views (true AP and axillary) were found to have lower accuracy and reliability in calculating glenoid bone loss (Table 1) [18, 42, 44, 45, 47]. Special radiographic views on the other hand, in particular the Bernageau profile view (Fig. 4), had better accuracy and reliability scores [18, 43, 46]. The Bernageau profile had an intra-observer intraclass correlation coefficient (ICC) 0.897-0.965 and inter-observer ICC 0.76-0.81 compared to a true AP radiograph with an intra-observer ICC 0.66-0.93 and inter-observer ICC 0.44-0.47 [43, 47]. Sommaire *et al.* demonstrated failure of Bankart repair when the width loss was 5.3 % compared to no failure with width loss 4.2 % using the Bernageau view (p = 0.003), although no clinically relevant threshold was defined [46]. A true AP radiograph was found to be specific (100 %) but not sensitive (54-65 %) for detecting a large glenoid bone lesion [45].

### CT and MRI

#### Surface area loss measurements

The surface area loss methods vary in the manner to which a circle is approximated to the inferior glenoid - based on either contralateral glenoid or intact posteroinferior margins of injured glenoid - but will be grouped together in this review as best-fit circle surface area methods (Fig. 10) (BCSA). The Pico Method (Fig. 2) is a BCSA based on the contralateral glenoid described by Baudi *et al*. [75]*.* The Pico Method has demonstrated good inter-observer (ICC 0.90) and intra-observer (ICC 0.94, 0.96-1.0) reliability as well as a low coefficient of variation (2.2-2.5 %) [32, 60, 65]. Magarelli *et al.* (mean difference between two-dimensional CT [2DCT] and three-dimensional CT [3DCT] 0.62 %+/−1.96 %) showed the method could be applied accurately with both 2DCT and 3DCT [32]. The previous article used software to calculate the surface area lost, but Barchillon *et al.* demonstrated that a BCSA method similar to the Pico Method could be applied using 3DCT, a femoral head gauge, and applying a mathematical formula [20]. Milano *et al.* demonstrated that using the Pico Method, recurrent dislocation was associated with defects greater than 20 %. [55] Huijsmans *et al.* showed that a BCSA method could be used by MRI with similar accuracy to 3DCT [21]. Nofsinger *et al.* used a method termed the Anatomic Glenoid Index (see Table 2 for description) in an attempt to retrospectively predict which surgical procedure an instability patient would receive but were unable to separate the two groups with their method [35].

#### Width loss measurements

**Fig. 5:**
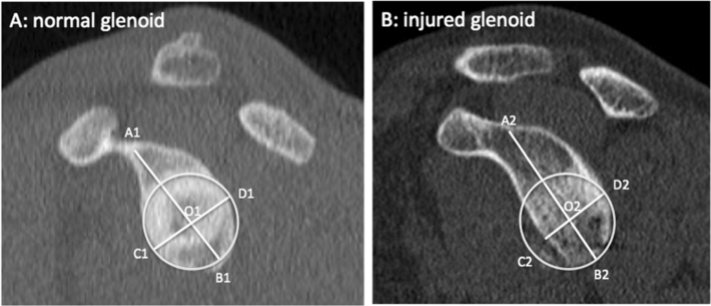
Glenoid Index. The Glenoid Index is calculated from injured width/normal width. Chuang *et al.* use the parameters of the normal glenoid to normalize the pre-injury glenoid width accounting for any height difference between shoulders. They then compare the ratio of post-injury width to pre-injury width. Although demonstrated here using 2D CT, the description of the Glenoid Index by Chuang *et al.* involves 3DCT. (Adapted from Chuang *et al.* [68])

Griffith *et al.* created the Griffith Index using bilateral shoulder CT scans (Fig. 1) as one of the first width loss techniques and it has been found to be reliable and accurate [33, 47, 58]. A method similar to the Griffith Index, termed the Glenoid Index (Fig. 5) was shown to accurately predict if a patient would require a Latarjet procedure 96 % of the time [68]. Other width loss measurement methods have measured width loss from a circle approximated to the inferior glenoid (based on either contralateral or ipsilateral glenoid) and have been found to have good reliability and accuracy (Table 3) [47, 48, 50, 52]. Evidence is conflicting with respect to whether width loss measurements can be applied to MRI. Tian *et al.* suggested volumetric interpolated breath-hold examination (VIBE) magnetic resonance arthrography (MRA) was as accurate as 2DCT and Lee *et al.* found a correlation between 2DCT and MRI of r = 0.83 and the difference in accuracy was 1.3 % for width loss measurements [51, 52]. Moroder *et al.* found that MRI (35 % sensitive, 100 % specific) was not as sensitive as 3DCT (100 % sensitive, 100 % specific) for detecting a significant bone lesion [50]. Gyftopoulous *et al.* found 3DCT was more accurate than 2DCT and MRI (percent error 3DCT 2.17-3.5 %, 2DCT 2.22-17.1 %, MRI 2.06-5.94 %) although the difference was not significant and they concluded that MRI could accurately measure glenoid bone loss [48].

#### Comparative studies

A few studies directly compared imaging modalities and methods (Table 4). Bishop *et al.* found 3DCT had the highest accuracy (agreement kappa 0.5) and second highest intra-observer reliability (intra-observer kappa 0.59) while radiography (agreement kappa 0.15, intra-observer kappa 0.45) had the lowest in a comparison of radiography, 2DCT, 3DCT, and MRI [42]. Rerko *et al.* also found that 3DCT was the most accurate (percent error [PE] -3.3 % +/− 6.6 %) and reliable (inter-observer ICC 0.87-0.93) method compared to radiography (PE −6.9 %; ICC 0.12-0.53), 2DCT (PE −3.7 %; ICC 0.82-0.89), and MRI (PE −2.75 %; ICC 0.38-0.85) [44]. Bois *et al.* evaluated 7 measurement techniques using 2DCT and 3DCT and found that the Pico Method based on the contralateral, intact glenoid (inter-observer ICC 0.84; PE 7.32) and Glenoid Index using 3DCT (ICC 0.69; PE 0.01) were the most reliable and accurate methods [63].

#### Other glenoid bone loss methods

**Fig. 6:**
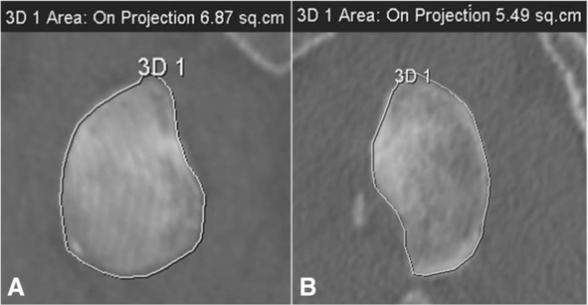
Glenoid bone loss calculated with De Filippo method using CT curved MPR. Normal right glena (**a**; 6.87 sq. cm), left glena with deficiency (**b**; 5.49 sq. cm)

**Fig. 7:**
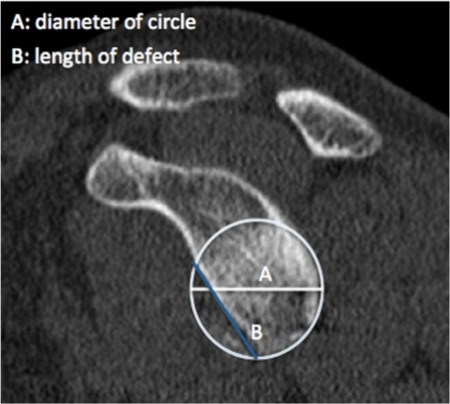
Gerber Index. The Gerber Index calculates bone loss based on a ratio of length of glenoid defect and diameter of glenoid. (Adapted from Sommaire *et al.* [46])

Results are presented in Table 5. Dumont *et al.* used an arc angle to determine percent surface area loss but have not tested the method clinically [49]. De Filipo *et al.* created a new technique using curved multiplanar reconstruction (MPR), a CT technique generally used for vascular studies for its ability to follow curved surfaces, and found it was more accurate than flat MPR in measuring surface area loss (Fig. 6) [66]. Diederichs *et al.* expressed bone loss in terms of volume using 3D computer software to mirror the contralateral normal glenoid and found good accuracy in a cadaveric study [59]. Sommaire *et al.* used Gerber’s Index and found that a threshold of 40 % bone loss was associated with recurrent instability (Fig. 7) [46].

#### Hill-Sachs imaging

##### Radiography

**Fig. 8:**
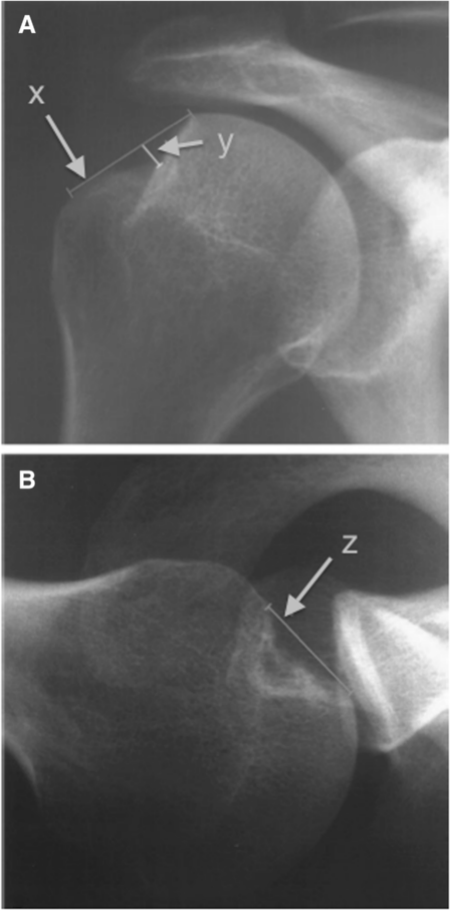
Hill-Sachs Quotient. **a)** True AP x-ray of the humerus with the shoulder in 60° internal rotation to measure the width (x) and depth (y) of the lesion. **b)** Bernageau profile view to measure the length (z) of the lesion. The Hill-Sachs Quotient is calculated by multiplying x, y and z. Grade: I <1.5 cm^2^; II 1.5-2.5 cm^2^; III > 2.5 cm^2^ (Adapted from Kralinger *et al.* [39])

Results are presented in (Table 6). Ito *et al.* positioned patients supine with the shoulder in 135 ° flexion and 15 ° of internal rotation to obtain a view of the posterolateral notch and calculate depth and width of the Hill-Sachs lesion but reliability was not explored [38]. Kralinger *et al.* calculated a Hill-Sachs Quotient (Fig. 8) by measuring depth and width on a true AP radiograph with the arm in 60 ° internal rotation and length on a Bernageau profile [39]. Recurrence rate was noted to be higher with a larger quotient (Grade I 23.3 %; grade II 16.2 %; grade III 66.7 %) although the reliability and accuracy have not been tested.

**Fig. 9:**
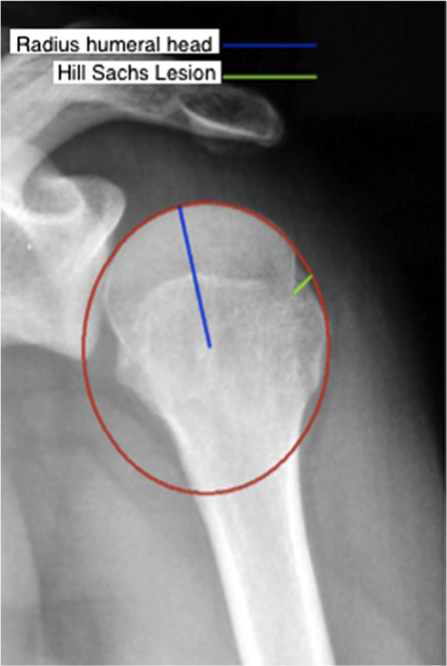
Humeral head depth:radius ratio (d/R). On a true AP x-ray with internal rotation, a circle template is fit to the contour of the articular surface of the humeral head and the depth of Hill-Sachs bone loss is measured. (Adapted from Sommaire *et al.* [46])

A technique of creating a ratio using the Hill-Sachs defect depth and humeral head radius (d/R) on a true AP radiograph with the arm in internal rotation has been found to be reliable and clinically relevant (Fig. 9). Charousset *et al.* demonstrated inter- and intra-observer reliability ICC 0.81-0.92 and 0.72-0.97 respectively with this technique [47]. Sommaire *et al.* retrospectively found the recurrence rate of GHI was 40 % when d/R >20 % compared to 9.6 % when d/R <20 %, while Hardy *et al.* found arthroscopic stabilization failure rate was 56 % when d/R > 15 % compared to 16 % when d/R <15 % [37, 46].

**Fig. 10:**
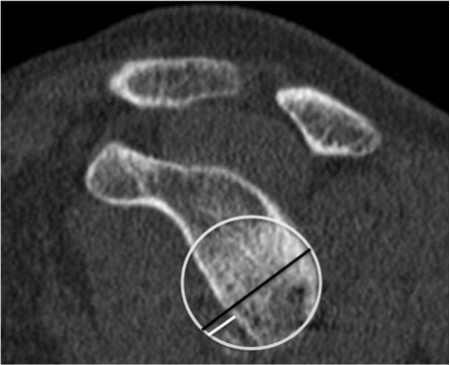
Best-Fit Circle Width Loss. A circle is approximated to the inferior glenoid and the expected diameter of the circle is compared to the defect width

### CT and MRI

Hardy *et al.* measured the volume of the defect on axial slices using width, depth, and length and found a significantly larger defect volume in patients with lower Duplay functional scores but did not evaluate reliability [37]. Saito *et al.* and Cho *et al.* both measured defect depth and width on the axial slice where the lesion was largest [23, 36]. Saito *et al.* found intra-observer Pearson correlation coefficients of 0.954-0.998 using 2DCT. Cho *et al.* found inter- and intra-observer reliability ICC of 0.772-0.996 and 0.916-0.999 respectively for depth and width measurements on 2DCT. Cho *et al.* also showed that the size of engaging Hill-Sachs lesions were significantly larger than non-engaging lesions. Kodali *et al.* evaluated the reliability and accuracy of 2DCT with an anatomic model and showed inter-observer reliability ICC 0.721 and 0.879 for width and depth respectively. The accuracy was highest in axial slices but there was still a percent error of 13.6+/−8.4 % [72].

Two studies evaluated MRI for quantifying Hill-Sachs lesions. Salomonsson *et al.* measured the depth of the lesion but were not able to show a significant difference between stable and unstable shoulders that were treated non-operatively [71]. Kirkley *et al.* tested the agreement between MRI and arthroscopy for the quantification of Hill-Sachs size [70]. They showed moderate agreement (Cohen’s kappa value 0.444) for the size of the lesion as normal, less than 1 cm, or greater than 1 cm.

## Discussion

Glenohumeral bone loss is a key factor in predicting recurrent instability following traumatic anterior shoulder dislocation. Increasing size of glenoid and Hill-Sachs lesions are associated with higher failure rates of arthroscopic Bankart repairs. Clinical and biomechanical studies have attempted to determine threshold values of glenohumeral bone loss to use in preoperative planning. Radiography, CT, and MRI have all been explored as imaging modalities for quantification of these glenohumeral bone lesions, with CT being studied most extensively. Within each modality a number of methods have been proposed to quantify the bone loss. There is still a need for further investigation to determine the best modality and method but significant progress has been made since 2000.

Radiography appears to have a role in screening patients for glenoid bone loss; standard radiographic views are straightforward for imaging technicians to obtain but their accuracy is low compared to CT and MRI [42]. Specialized radiographic views are more accurate; however they may be difficult to reproduce clinically due to patient discomfort or apprehension [74]. Slight deviations in gantry orientation or arm positioning may also obscure the bony details [47].

Two general methods emerged when quantifying glenoid bone loss using advanced imaging, width and surface area methods. Each of these methods uses humeral head subtraction and reconstruction to obtain *en face* views of the glenoid. Because bone loss occurs anteriorly, width methods measure the bone loss in this anterior-posterior dimension in the inferior 2/3 of the glenoid. Ji *et al.* state that this measurement should be made specifically at 03:20 on a clock face, the most common location of bone loss [13]. The bone loss in the width methods is expressed compared to the pre-injury glenoid. Some authors have used CT scans of the contralateral, uninjured shoulder to obtain the pre-injury width. Others have approximated the pre-injury width by using the intact inferior edges of the injured glenoid to place a circle. Earlier studies suggested that either form of calculating the width loss was adequate, although newer studies have found that using bilateral CT is more accurate [42, 63]. The Glenoid Index (Fig. 5) is the most accurate width measurement method. Surface area methods function by approximating a circle to the inferior glenoid in the same manner as the width methods then finding the area missing. The surface area methods have been studied frequently and have good to excellent reliability [32, 60, 65]. Initially computer software was required to calculate the surface area; however Barchillon *et al.* showed that one could use a femoral gauge in a clinical setting and still produce accurate results [20]. Comparative studies suggest that the Pico Method based on bilateral CT scans is most accurate and reliable, particularly when using 3DCT versus 2DCT [42, 48, 63]. The accuracy of the Pico method may be affected in part due to the curved nature and concavity of the glenoid. De Felippo *et al.* have attempted to address this with their De Felippo method that utilizes curved MPR. This was shown to have low interobserver variability and a high correlation with their reference standard. Evidence is currently equivocal if MRI can be used to calculate glenoid bone loss using width and surface area methods in a clinical setting [48, 50, 61]. In comparative studies, CT was found to be more accurate than MRI [42, 48].

Radiography has been shown to be useful in quantifying Hill-Sachs bone loss. Creating a depth/radius ratio on a true AP radiograph with internal rotation is reliable and shown to be clinically relevant, although the accuracy has not been clarified [37, 46, 47]. CT imaging has been used to quantify depth, width, and volume of the lesion. It appears the reliability of CT is good but its accuracy is questionable, often underestimating the size of the lesion [72]. A complicating factor in determining the significance of a Hill-Sachs lesion is the role its orientation plays. More horizontal lesions tend to engage the glenoid lesion and incorporating this factor along with the size is likely important but has not been determined yet [36]. Walia *et al.* have explored how Hill-Sachs and glenoid lesions interact and engage theoretically and concluded that combined bony defects reduced stability more than expected based on isolated defects alone [30]. The exact way they reduce stability and how to calculate this clinically is unknown.

This study is limited by its qualitative nature. Because the literature has evaluated multiple methods and modalities with different statistical tools it is difficult to pool data and achieve a clear answer of which method is the best. However, this may guide future prospective studies into which method to apply. There was a trend to use either a width or surface-area method, each of which can be applied with the same imaging data set obtained from a CT or MRI. Comparing these methods and correlating with surgical results may give an answer to the best imaging method.

## Conclusions

Our scoping review has synthesized the current evidence regarding imaging techniques to quantify glenoid and Hill-Sachs bone loss. A number of modalities and methods have been explored to quantify glenoid bone loss. Radiography does not appear to have the accuracy required for pre-operative planning but may play a role in screening patients that would require advanced imaging. The Bernageau profile view to calculate AP glenoid width is the most accurate radiograph. CT is the most accurate modality but the risk of radiation exposure, particularly when using methods that require bilateral imaging, needs to be considered. Of the methods used with CT, the Glenoid Index is the most accurate and reliable width method while the Pico Method is the most accurate and reliable surface area method. The Glenoid Index requires bilateral shoulder CT and the Pico Method is most accurate when applied using bilateral CT but may also be applied with unilateral imaging. There are equivocal findings about the accuracy or MRI compared to CT and this needs to be clarified by future studies.

A consensus measurement technique for calculating Hill-Sachs bone loss or a threshold size for predicting recurrent instability has not yet been established. Larger lesions, at least >25 % of the humeral head diameter, appear to increase risk of recurrent instability in biomechanical studies [24]. Calculating a depth:radius radio on a true AP radiograph with the arm internally rotated is inexpensive, easy to obtain, and predicts recurrence with good reliability when >20 %. However, its accuracy has not yet been established. Measuring the depth and width on axial slices of a CT scan have good to excellent reliability and have been associated with engaging Hill-Sachs lesions. However the role of CT in predicting recurrence has not been determined.

Ease of calculation, radiation exposure, experience of interpreting radiologist or surgeon, and software availability are factors that should be considered when determining which method will be used. Finally, glenoid and Hill-Sachs bone loss may need to be evaluated together as the manner in which these lesions interact is complex and requires further study.

## Additional file

Additional file 1:
**Appendix 1_BMC.docx; This is a Microsoft Word file that contains the Search Strategy for the scoping review.**

## Abbreviations

2DCT: Two-dimensional computed tomography
3DCT: Three-dimensional computed tomography
BCSA: Best-fit circle surface area
d/R: Hill Sachs defect depth and humeral head radius
CT: Computed tomography
GHI: Glenohumeral instability
ICC: Intraclass correlation coefficient
MPR: Multiplanar reconstruction
MRA: Magnetic resonance arthrography
MRI: Magnetic resonance imaging
PE: Percentage error
VIBE: Volumetric interpolated breath-hold examination
