# Supplementary material for: Imaging methods for quantifying glenoid and Hill-Sachs bone loss in traumatic instability of the shoulder: a scoping review
Source: BMC Musculoskelet Disord. 2015 Jul 18;16:164. doi: 10.1186/s12891-015-0607-1 (PMC4506419; doi:10.1186/s12891-015-0607-1)
Supplement: Additional file 1: — Appendix 1_BMC.docx; This is a Microsoft Word file that contains the Search Strategy for the scoping review. [file 12891_2015_607_MOESM1_ESM.pdf]

Appendix

**MEDLINE Search Strategy**	
1	(computed tomography or ct).mp.
2	image interpretation, computer-assisted/ or radiographic image interpretation, computer-assisted/ or exp tomography, emission-computed/ or tomography, x-ray computed/ or four-dimensional computed tomography/ or tomography, spiral computed/ or imaging, three-dimensional/ or holography/
3	exp Radiography/
4	X-Rays/
5	(radiograph* or x ray*).mp.
6	exp Magnetic Resonance Imaging/
7	(magnetic resonance or mri).mp.
8	image processing, computer-assisted/ or imaging, three-dimensional/
9	Diagnostic Imaging/
10	imaging.ti.
11	or/1-10
12	Shoulder Joint/
13	Humeral Head/
14	(glenoid or hill sachs or bankert or glenohumeral or humeral head).mp.
15	12 or 13 or 14
16	Joint Instability/
17	shoulder dislocation/ or shoulder fracture/
18	((bony or bone) and (defect* or lesion* or loss or fracture*)).mp.
19	16 or 17 or 18
20	11 and 15 and 19
21	di.fs.
22	diagnos*.mp.
23	exp "Sensitivity and Specificity"/
24	(inter rater or interrater or intrarater or intra rater).mp.
25	(sensitiv* or specific*).mp.
26	accuracy.ti,ab.
27	(quantif* or measur*).ti,ab.
28	21 or 22 or 23 or 24 or 25 or 26 or 27
29	20 and 28
30	((glenoid or hill sachs or bankert or glenohumeral or humeral head) adj5 (defect or loss or lesion* or fracture*)).mp.

31	11 and 30
32	29 or 31
**EMBASE Search Strategy**	
1	radiography/ or exp shoulder radiography/
2	2. computer assisted diagnosis/ or computer assisted radiography/ or exp computer assisted tomography/
3	3. X ray/
4	4. (x ray or radiograph* or computed tomography or ct).ti.
5	5. (magnetic resonance imaging or magnetic resonance arthrography or mri).ti.
6	6. radiodiagnosis/ or diagnostic imaging/ or exp nuclear magnetic resonance imaging/ or exp tomography/
7	7. or/1-6
8	8. Joint Instability/
9	9. ((bony or bone) and (defect* or lesion* or loss or fracture*)).ti,ab. or osteolysis/
10	10. shoulder dislocation/ or shoulder fracture/
11	11. shoulder/
12	12. humerus head/
13	13. (glenoid or hill sachs or bankert or glenohumeral or humeral head).mp.
14	14. 11 or 12 or 13
15	15. 8 or 9 or 10
16	16. 7 and 14 and 15
17	17. di.fs.
18	18. predict.ti,ab.
19	19. specificity.ti,ab.
20	20. diagnos*.ti,ab.
21	21. (quantif* or measur*).ti,ab.
22	22. 17 or 18 or 19 or 20 or 21
23	23. 16 and 22
24	24. ((glenoid or hill sachs or bankert or glenohumeral or humeral head) adj5 (defect or loss or lesion* or fracture*)).mp.
25	25. 7 and 24
26	26. 23 or 25
**Scopus Search Strategy**	
((TITLE((glenoid OR "hillsachs" OR shoulder OR bankart)) AND TITLE((fracture* OR loss O R instability OR defect* OR lesion*)) AND TITLE(imaging OR roentgenogram* OR radiolog* OR xray* OR ct OR "computed tomography" OR mri OR "magnetic resonance")))OR (((TITLE((glenoid OR "hill sachs" OR shoulder OR bankart)) OR KEY((glenoid OR "hillsachs" OR shoulder OR bankart)))) AND ((TITLE(fracture* OR loss OR instability OR defect* OR lesion*) OR KEY(fracture* OR	

loss OR instability OR defect* OR lesion*))) AND((TITLE(bone) OR KEY(bone))) AND ((TITLE(imaging OR roentgenogram* OR radiolog* OR x-ray* OR ct OR "computed tomography" OR mri OR "magnetic resonance") OR KEY(imaging OR roentgenogram* OR radiolog* OR x-ray* OR ct OR "computed tomography" OR mri OR"magnetic resonance"))))
**Web of Science Search Strategy**

# 3	**687**	#2 OR #1<br><br>*Databases=SCI-EXPANDED, CPCI-S*<br>*Timespan=All Years*<br>*Lemmatization=On*
# 2	**611**	Topic=((glenoid or "hill sachs" or shoulder or bankart)) AND Topic=((fracture* or loss or instability or defect* or lesion*) AND bone) AND Topic=(imaging or roentgenogram* or radiolog* or x-ray* or CT or "computed tomography" or mri or "magnetic resonance")<br><br>*Databases=SCI-EXPANDED, CPCI-S*<br>*Timespan=All Years*<br>*Lemmatization=On*
# 1	**103**	Title=((glenoid or "hill sachs" or shoulder or bankart)) AND Title=((fracture* or loss or instability or defect* or lesion*)) AND Title=(imaging or roentgenogram* or radiolog* or x-ray* or CT or "computed tomography" or mri or "magnetic resonance")<br><br>*Databases=SCI-EXPANDED, CPCI-S*<br>*Timespan=All Years*<br>*Lemmatization=On*
